# Evolocumab Treatment in Dyslipidemic Patients Undergoing Coronary Artery Bypass Grafting: One-Year Safety and Efficacy Results

**DOI:** 10.3390/jcm13102987

**Published:** 2024-05-19

**Authors:** Giuseppe Nasso, Walter Vignaroli, Vincenzo Amodeo, Francesco Bartolomucci, Claudio Larosa, Gaetano Contegiacomo, Maria Antonietta Demola, Cataldo Girasoli, Antongiulio Valenzano, Flavio Fiore, Raffaele Bonifazi, Vera Triggiani, Vincenza Vitobello, Giacomo Errico, Angela Lamanna, Dritan Hila, Tommaso Loizzo, Rosalba Franchino, Stefano Sechi, Giovanni Valenti, Giuseppe Diaferia, Mario Siro Brigiani, Serena Arima, Mario Angelelli, Antonio Curcio, Francesco Greco, Ernesto Greco, Giuseppe Speziale, Giuseppe Santarpino

**Affiliations:** 1 Department of Cardiac Surgery, Anthea Hospital, GVM Care & Research, 70124 Bari, Italy; gcontegiacomo@gvmnet.it (G.C.); mariaantoniettademola@gmail.com (M.A.D.); cataldo.girasoli@gmail.com (C.G.); antongiulio.valenzano@hotmail.it (A.V.); ffiore@gvmnet.it (F.F.); bgiava@tiscali.it (R.B.); veratriggiani@libero.it (V.T.); vincenzavitobello@gmail.com (V.V.); giacomo.errico1@gmail.com (G.E.); an.lamanna@gmail.com (A.L.); dritan.hila@gmail.com (D.H.); tloizzo@gvmnet.it (T.L.); rfranchino@gvmnet.it (R.F.); msirobrigiani@gvmnet.it (M.S.B.); gspeziale@gvmnet.it (G.S.); 2 Department of Cardiac Surgery, San Carlo di Nancy, GVM Care & Research, 00137 Rome, Italy; vignaroli.walter@gmail.com (W.V.); ssechi88@gmail.com (S.S.); 3 Department of Cardiology, “Santa Maria degli Ungheresi” Hospital, 89024 Polistena, Italy; enzoamodeo55@libero.it; 4 Department of Cardiology Azienda Ospedaliera B.A.T., Bonomo Hospital, 70031 Andria, Italy; f.bartolomucci@aslbat.it (F.B.); larosa.cld@gmail.com (C.L.); gv1269@gmail.com (G.V.); 5 Department of Cardiology, “M. Di Miccoli” Hospital, 70051 Barletta, Italy; gdiaferia@alice.it; 6 Department of Human and Social Sciences Unisalento, University of Salento, 73100 Lecce, Italy; serena.arima@unisalento.it (S.A.); mario.angelelli@unisalento.it (M.A.); 7 Division of Cardiology, Department of Pharmacy, Health and Nutritional Science, University of Calabria, 87036 Rende, Italy; antonio.curcio.cardio@unical.it; 8 Department of Cardiology, “Santissima Annunziata” Hospital, 87100 Cosenza, Italy; francesco.greco@aocs.it; 9 Department of Clinical, Internal Medicine, Anesthesiology and Cardiovascular Sciences, Sapienza University of Rome, 00185 Rome, Italy; ernesto.greco@uniroma1.it; 10 Department of Clinical and Experimental Medicine, Magna Graecia University, 88100 Catanzaro, Italy; gsantarpino@gvmnet.it; 11 Department of Cardiac Surgery, Città di Lecce Hospital, GVM Care & Research, 73100 Lecce, Italy; 12 Department of Cardiac Surgery, Paracelsus Medical University, 90419 Nuremberg, Germany

**Keywords:** coronary artery bypass grafting, dyslipidemia, atherosclerosis

## Abstract

**Background:** The inhibition of PCSK9 lowered LDL cholesterol levels, reducing the risk of cardiovascular events. However, the effect on patients who have undergone surgical myocardial revascularization has not yet been evaluated. **Methods:** From January 2017 to December 2022, 180 dyslipidemic patients who underwent coronary artery bypass were included in the study. Until December 2019, 100 patients optimized therapy with statin ± ezetimibe (SG). Since January 2020, 80 matched patients added treatment with Evolocumab every 2 weeks (EG). All 180 patients were followed-up at 3 and 12 months, comparing outcomes. **Results:** The two groups are homogenous. At 3 months and 1 year, a significant decrease in the parameter mean levels of LDL cholesterol and total cholesterol is detected in the Evolocumab group compared to the standard group. No mortality was detected in either group. No complications or drug discontinuation were recorded. In the SG group, five patients (5%) suffered a myocardial infarction during the 1-year follow-up. In the EG group, two patients (2.5%) underwent PTCA due to myocardial infarction. There is no significant difference in overall survival according to the new treatment (*p*-value = 0.9), and the hazard ratio is equal to 0.94 (95% C.I.: [0.16–5.43]; *p*-value = 0.9397). **Conclusions:** The use of Evolocumab, which was started immediately after coronary artery bypass graft surgery, significantly reduced LDL cholesterol and total cholesterol levels compared to statin treatment alone and is completely safe. However, at one year of follow-up, this result did not have impact on the reduction in major clinical events.

## 1. Introduction

Research has firmly established that low-density lipoprotein (LDL) cholesterol is a modifiable risk factor for cardiovascular disease. Monoclonal antibodies target proprotein convertase subtilisin-kexin type 9 (PCSK9) [1], and among these, Evolocumab, a fully human monoclonal antibody, significantly reduces LDL cholesterol levels by approximately 60% [2,3,4,5,6]. The medical literature regarding genetic basis has demonstrated that patients with PCSK9 loss-of-function alleles exhibit lower LDL cholesterol levels, leading to a consequent decrease in the risk of myocardial infarction [7,8]. Prolonged observations in phase 2 and phase 3 trials examining PCSK9 inhibitors demonstrated noteworthy declines in cardiovascular complications, but the findings in these studies did not exceed 100 [9,10]. The Further Cardiovascular Outcomes Research with PCSK9 Inhibition in Subjects with Elevated Risk (FOURIER) clinical trial was specifically designed to assess cardiovascular outcomes, evaluating the clinical effectiveness and safety of Evolocumab when administered alongside high-intensity or moderate-intensity statin therapy in patients manifesting clinically evident atherosclerotic cardiovascular disease. LDL cholesterol levels lowered to a median of 30 mg per deciliter (0.78 mmol per liter), reducing the risk of cardiovascular incidents inhibiting PCSK9 with Evolocumab and statin therapy [9]. These results emphasize the advantages of reducing LDL cholesterol levels below prevailing targets for individuals with atherosclerotic cardiovascular disease.

Despite advancements in cardiopulmonary support devices and surgical techniques, myocardial injury during cardiac surgery, associated with ischemia and reperfusion, remains inevitable, with reported rates approximately 5–15% higher in patients who have undergone coronary artery bypass grafting (CABG) [11,12,13,14,15]. The occurrence of myocardial injury during surgery is associated with significant complications, resulting in elevated rates of morbidity, mortality, and hospital expenditure [16]. The processes underlying ischemia and reperfusion injury during surgical procedures are intricate. Myocardial stunning and microvascular dysfunction are possible causes by direct ischemia, thrombosis, oxygen free radicals, inflammation, endothelial dysfunction, and changes in cellular metabolism [17]. To investigate and mitigate myocardial injury during coronary artery bypass surgery, there have been numerous clinical trials. Presently, statins remain the medication for clinical application [18,19,20]. However, the ongoing debate persists, with several ongoing studies [21,22]. Beyond their primary function of lowering LDL cholesterol levels, statins, hydroxymethyl-glutaryl-coenzyme A (HMG-CoA) reductase inhibitors, also contribute to cardioprotection through their anti-inflammatory, antioxidant, and immunomodulatory properties [23,24,25]. The effects of PCSK9 inhibitors extend to diverse clinical applications beyond cholesterol reduction. Through their distribution along arterial vascular walls, PCSK9 inhibitors demonstrate anti-inflammatory effects, thereby reducing inflammation and atherosclerosis [9,26,27,28,29]. PCSK9 inhibitors may have immunoregulatory properties, with findings from septic shock models suggesting potential benefits from their use [30]. Administering PCSK9 before surgery reduces ischemic and reperfusion injury to the myocardium, resulting in enhanced cardiac function [31]. PCSK9 inhibitors could offer advantages to patients recovering from cardiovascular surgery due to their cardioprotective effects. The efficacy of PCSK9 inhibitors remains unproven. This study aims to assess the efficacy and safety of Evolocumab in patients undergoing elective coronary artery bypass grafting, initiating treatment one-month post-operation in individuals unresponsive to standard therapy. Presented here are our preliminary findings.

### Ethical Statement

The present study conforms to the Declaration of Helsinki. It was approved by our institutional review board for human research for retrospective studies (Prot. Number. 63/U/2023) and registered on 22 October 2023 (I.D. study 865; code number AH.19.11.2023) to the observational registry for clinical trials of “Italian Agency of Medicines” (AIFA—Agenzia Italiana del Farmaco).

On 6 February 2024, the local ethical committee approved the study (Prot. N. 001.06.96. Study number 7815).

Patients consented for their clinical data to be used for research.

## 2. Methods

From January 2017 to December 2022, at the cardiac surgery of Anthea Hospital GVM Care & Research Bari, 592 patients (498 men and 94 women) were operated on with elective isolated coronary surgery due to stable or atypical angina and coronary angiography study performed electively. Of these patients, 230 (39%) were affected by dyslipidemia while being preoperatively treated with statins or not receiving cholesterol-lowering treatment at all. Among these dyslipidemic operated patients, 180 were seen one month after the operation and at the following follow-up by the cardiac surgeon, who evaluated the therapy adhered to by the patient and the laboratory tests, including the lipid profile.

**Figure 1:**
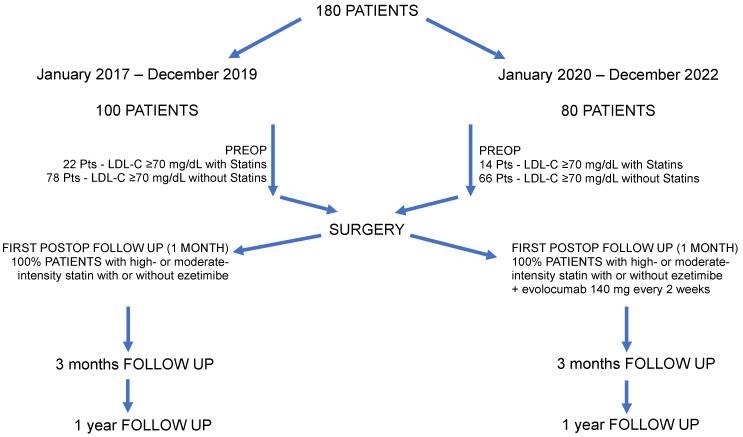
Study timeline.

Until December 2019, 100 patients with these characteristics were seen, and of these, 22 had an LDL-C concentration ≥ 70 mg/dL while on optimized lipid-lowering therapy, including a high- or moderate-intensity statin with or without ezetimibe, and 78 patients had LDL-C concentrations ≥ 70 mg/dL without statin therapy. Since January 2020, once Evolocumab was usable in patients with high LDL-C values, the next 80 patients with the same characteristics as the previous ones began statin therapy with or without ezetimibe, as well as treatment with 140 mg subcutaneous Evolocumab every 2 weeks (Figure 1).

We started Evolocumab therapy after one month to reassess patients’ cholesterol levels and to be sure of treatment with Evolocumab.

These patients were followed for three months and one year at a clinical and laboratory level, and the data were compared between the 2 groups, in particular, the results were compared in terms of the ability to have an effect on the laboratory values of LDL-C and clinically for the risk of cardiovascular disease in the field of secondary prevention. All adverse events were analyzed, but, in particular, the risks of recurrent angina, myocardial infarction, cerebrovascular events, need for coronary re-angiography, and coronary artery re-angioplasty/re-bypass were analyzed. At follow-up checks, in all patients, the Alanine Transaminases were also checked.

## 3. Statistical Analysis

The results are expressed as mean and standard deviation (SD) for continuous variables and as frequencies for categorical variables. A comparative analysis between subjects undertaking new and standard treatment was performed based on each variable of interest. Variables were checked for normality by means of the Kolmogorov–Smirnov test for normal distribution and normality was accepted when *p* ≤ 0.05. The χ^2^-squared was used for categorical variables, while the two-sample Student’s *t*-test or Wilcoxon test was considered for continuous variables. We fixed a significance level equal to 0.05. Body mass index was calculated as weight/height^2^ (kg/m^2^) and treated as a continuous variable.

To avoid the potential bias of covariates not evenly distributed between the patients treated with standard treatment and those with the new one, propensity score (PS) matching was performed using the open-source R software (version 4.3.3). This procedure is frequently used in quasi-experimental studies to generate an unbiased control group, enabling the exploration of causal relationships using observational data [32]. In our study, the PS was based on demographic variables (age, sex, and socioeconomic status), as well as on factors that are known to affect LDL and total cholesterol (smoking, BMI, high blood pressure, and diabetes mellitus). Using the Match In package in R, we evaluated the unbalancing factor in the treatment allocation. Looking at the standardized mean differences, variance ratios, and empirical cumulative distribution function (eCDF) statistics, we cannot detect severe imbalances. Indeed, the values of standardized mean differences and eCDF statistics are close to 0, and the values of variance ratios are close to 1, indicating good balance and no need for PS correction.

## 4. Results

**Figure 2:**
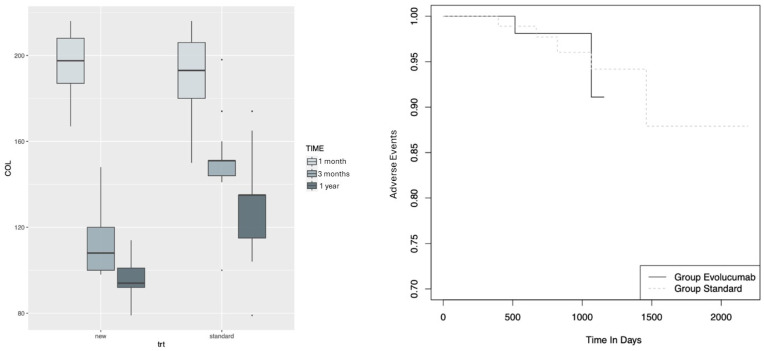
(**Left panel**): Distribution of the cholesterol at different time points (post-op: 1 month, time 1: 3 months, time 2: 1 year) for the two treatment groups. (**Right panel**): Time-to-adverse event for the two treatments.

Table 1 shows summaries of patients’ parameters in the two groups, namely, standard treatment and new treatment. The two groups are homogenous since none of the individuals’ parameters (such as gender, age, smoking habits, etc.) are significantly different between the two groups. We also report the average measurements of total cholesterol, LDL cholesterol, HDL cholesterol, Triglycerides, and Alanine at the three-time points and compare their values in the two groups. At baseline (Time 0—post-op), all parameters, except for Alanine Transaminase, are not significantly different in the two groups. However, at time 1 (3 months) and time 2 (1 year), a significant decrease in the parameter mean levels is detected in the group undertaking the new treatment with respect to the other group. The same decrease is observed for Alanine Transaminase, although the patients are significantly different even at baseline. Figure 2 (left panel) shows the distribution of cholesterol at different time points for the two treatment groups.

We used a mixed-lines model to evaluate the variation in the LDL and total cholesterol levels over time, comparing the trend in the two treatments. This choice allows us to conduct an overall analysis while identifying the treatment effects at different times. An overall significant decrease in the mean LDL level is detected considering all patients; indeed, the LDL cholesterol decreases, on average, by 62 from the baseline to the first control and by another 29 from the first to the second control. Moreover, such a decrease is significantly higher for the new treatment; indeed, at time 1, patients who undertook the new treatment showed a mean LDL cholesterol reduction of 85 units (157 → 72 mg/dL), in comparison to those who undertook the standard treatment, who had a reduction of 41 (114 vs. 72 mg/dL) (*p*-value < 0.001). At time 2, the LDL cholesterol level continued to significantly decrease, with an average significant reduction of 28 (72 → 44 mg/dL) in patients treated with the new treatment compared to those treated with the standard one (114 vs. 84 mg/dL) (*p*-value < 0.001).

Similarly, an overall significant decrease in the mean total cholesterol level is detected; indeed, the total cholesterol decreases, on average, by 62 from the baseline to the first control and by 20 from the first to the second control. Moreover, such a decrease is significantly higher for the new treatment; indeed, at time 1, patients who undertook the new treatment showed a mean total cholesterol reduction of 84 (195 → 111 mg/dL), compared to those who undertook the standard treatment 39 (191 → 152 mg/dL) (*p*-value < 0.001). At time 2, the cholesterol level continued to significantly decrease, with an average significant reduction of 16 (111 → 95 mg/dL) in the patients treated with the new treatment with respect to those treated with the standard one 22 (152 → 130 mg/dL) (*p*-value < 0.001).

**Figure 3:**
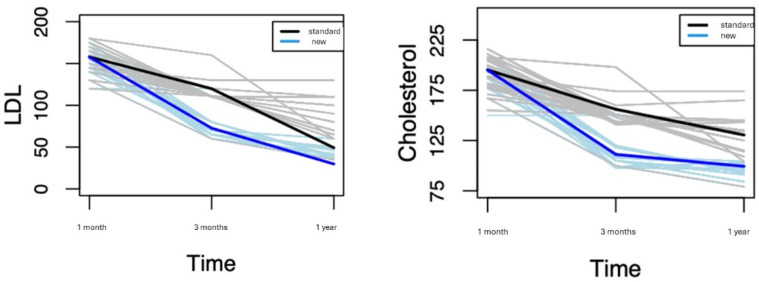
(**Left panel**): LDL measurements at different time points for the patients treated with standard treatment (grey lines) and “new” Evolocumab treatments (blue lines). The bold line are the regression line estimates. (**Right panel**): total cholesterol measurements at different time.

Figure 3 shows the LDL cholesterol and total cholesterol levels at each time point for the two treatment groups. Bold lines refer to the fitted regression model and represent the estimated average time trend of the LDL cholesterol (left panel) and total cholesterol (right panel).

No patients died at follow-up in both groups. No patients showed side effects (back pain, bleeding, blistering, burning, coldness, discoloration of the skin, feeling of pressure, hives, infection, inflammation). Ten patients in the standard group (10%) and three in the Evo group (3.7%) reported episodes of re-angina at follow-up. One patient in the standard (1%) and one in the Evo group (1.2%) needed hospitalization during the follow-up due to a cerebral transient ischemic attack. In the standard group, five patients (5%) suffered a myocardial infarction during the 1-year follow-up; four were treated with PTCA and one underwent re-CABG. In the Evo group, two patients (2.5%) underwent PTCA due to myocardial infarction at follow-up.

To evaluate the probability of adverse events (defined as myocardial infarction, need for re-CABG and re-PTCA, and stroke), we used a survival regression model. There is no significant difference in overall survival according to the Evolocumab treatment (*p*-value = 0.9), and the hazard ratio is equal to 0.94 (95% confidence interval: [0.16–5.43]), with a *p*-value equal to 0.9397.

## 5. Discussion

As we know, this is one of the first studies comparing effects of Evolocumab added to statin therapy analyzing only patients who underwent coronary artery bypass grafting [1,2].

In our research, we observed that adding the PCSK9 inhibitor Evolocumab to statin therapy resulted in a reduction in LDL cholesterol levels from 158 mg/dL to 44 mg/dL from preoperative measurements to the 1-year follow-up, as compared to patients solely on statin therapy. This effect persists from 3 months postoperatively to 1 year of follow-up. At the one-year follow-up, the sole adverse events observed with Evolocumab were rare injection-site reactions. There was no statistically significant difference in the occurrence of complications, such as myocardial infarction, repeat percutaneous transluminal coronary angioplasty (re-PTCA), and repeat coronary artery bypass grafting (re-CABG), compared to statin therapy. On this clinical consequence, the results of our study differ from the Fourier trial [9]; they reported a significant reduction in the risk of cardiovascular events with the addition of Evolocumab to statin therapy—a 15% decrease in the risk of cardiovascular death, myocardial infarction, stroke, hospitalization for unstable angina, or coronary revascularization. However, we highlight that these authors, compared with our study, monitored the patients for a longer follow-up (26 months of follow-up vs. 12 months in our analysis) [9]. Precisely because of the aforementioned study [9], the 2016 guidelines on the treatment of dyslipidemia have been changed by more clearly introducing the use of PCSK inhibitors, in particular for secondary prevention for patients at very-high risk of not achieving their goal on a maximum tolerated dose of statins and ezetimibe [11]. Our study was unable to achieve this result in order to be able to make this suggestion for patients undergoing coronary artery bypass grafting. However, patients undergoing surgical myocardial revascularization are already suffering from advanced ischemic heart disease and undergoing secondary prevention. Therefore, regardless of the results we obtained, the use of Evolocumab in patients who do not reach the therapeutic target with statins alone after revascularization treatment is correct—be it percutaneous or surgical.

The positive long-term effects of the addition of Evolocumab are, however, probably still only partially known given that the long-term results of the therapy in patients at cardiovascular risk have also been published, demonstrating that the long-term reduction in LDL-C with Evolocumab was associated with persistent low rates of adverse events that did not exceed those observed in the original placebo arm [33]. Furthermore, this same study demonstrated that if Evolocumab was subsequently inserted into “placebo” patients, the protective effect was still reduced compared to patients who had taken it “immediately” [33]. Therefore, also in support of our study, it appears important to underline the early intake of the drug itself, as proposed by us in our immediate approach, i.e., one month after the surgical revascularization procedure. On the other hand, an early start is a hypothesis on which our study is based and has had clear bibliographical support for several years. In fact, a lag between the initiation of LDL cholesterol reduction and the realization of the complete clinical benefits in terms of risk reduction has been reported [9,34,35,36,37].

The benefit of decreasing LDL cholesterol levels therefore remains indisputable, given that even studies prior to the use of Evolocumab showed significant reductions in major cardiovascular events using statins alone in the Pravastatin or Atorvastatin Evaluation and Infection Therapy–Thrombolysis in Myocardial Infarction (PROVE IT–TIMI) and Treating to New Targets (TNT) trials [38,39]. It is therefore fundamental to reduce the value itself, not how it is obtained. For example, in patients who were statin-resistant, the addition of ezetimibe to statin therapy in the Improved Reduction of Outcomes: Vytorin Efficacy International Trial (IMPROVE-IT) lowered LDL cholesterol levels from 70 mg per deciliter to 54 mg per deciliter and significantly reduced major cardiovascular events [40]. Our study therefore fits into this context of attempts to reduce LDL-C values in patients who did not use statins with or without ezetimibe. However, we recorded that comparing the values obtained with Evolocumab vs. statins with or without ezetimibe, the reduction in cholesterol values at 3 months and 1 year was significantly more important. It can therefore be speculated that even if a patient responds therapeutically to statins, with or without ezetimibe, the addition of Evolocumab has a faster and more significant effect on the reduction in cholesterol, without increasing the risk of side effects.

But how much must the LDL-C value be reduced to have an “impact”? In the FOURIER study, in which consistent reductions in rates of cardiovascular events were seen, the LDL cholesterol level was lowered from 126 mg per deciliter to 43 mg per deciliter [9]. In our study, we managed to obtain a similar decrease, bringing the average LDL-C values at one-month post-intervention from 158 mg per deciliter to 44 mg per deciliter at one year post-intervention. Moreover, also in the Global Assessment of Plaque Regression with a PCSK9 Antibody as Measured by Intravascular Ultrasound (GLAGOV) trial [41], cardiovascular advantages were obtained, even with LDL cholesterol levels lowered to 20 to 25 mg per deciliter. Precisely, this effect on the stabilization and reduction in the volume of the atherosclerotic plaque represents a further possible explanation for the positive effect obtained by the patients in our study, who had critical atherosclerotic plaques that required surgical revascularization but also had widespread subcritical conditions which did not increase during follow-up in such a way as to make them clinically manifest in a different way to what was recorded in the patients who did not receive Evolocumab. The various studies that have used Evolocumab to date have, however, raised alerts for possible problems. For example, the suspension rate of Evolocumab injections due to adverse events was not applicable, as all 80 patients continued the treatment for up to one year, even those who had skin reactions at the injection site. Furthermore, a risk of new-onset diabetes and an increased risk of neurocognitive adverse events was also assumed. Even for these potential complications, at one year, no non-diabetic patient had problems related to abnormal increases in blood sugar, nor did any patient develop onset of neurocognitive symptoms. Indeed, the rates of new-onset diabetes did tend to be somewhat higher in patients with very low LDL-C levels in other studies and, furthermore, a relationship between low LDL-C levels and diabetes has been variably observed in some but not all cross-sectional studies and in some long-term follow-up population cohorts, regardless of lipid-lowering therapy [42,43]. Moreover, the long-term efficacy and safety of achieving a very low LDL-C level are supported by data from cohort studies of individuals with genetic *PCSK9* inhibition. In these studies, subjects who have lifelong LDL-C levels of ~15 mg/dL are also well, without apparent health, learning, or reproductive concerns [7,44,45,46,47,48]. Only some early epidemiological studies have raised concerns that very low LDL-C may be associated with an increased risk of hemorrhagic stroke and neurocognitive effects [49]. However, that phenomenon is observed before the full onset of the clinical benefits of LDL-C lowering, as has been documented across all the major LDL-C-lowering drug classes [9,24,34,40]. The mean limitation of our study is the lack of a long term follow-up. However, to date, the availability of studies to examine the long-term safety and efficacy of PCSK9 inhibitors has been limited. The phase 3 FOURIER trial demonstrated that a reduction in LDL-C is not correlated with a reduction in cardiovascular death, which was not observed over a median of 2.2 years. One longer study, OSLER-1 (Open Label Study of Long-Term Evaluation Against LDL-C Trial), has shown that Evolocumab appears to be safe and well tolerated in patients with hypercholesterolemia, with a follow-up of up to 5 years. In the FOURIER-OLE study, long-term treatment with Evolocumab was studied in 6635 subjects, with a median follow-up of 5 years and maximum exposure times of >8 years when parent and extension studies were combined. However, in this study, the majority of subjects achieved very low LDL-C levels, achieving an LDL-C of <40 mg/dL at 12 weeks. This demonstrates that studies, such as that in our paper, without a long term follow-up also have an important clinical impact, in particular on the efficacy of reducing LDL-C. However, we support the importance of having trials focusing on cardiovascular outcomes of lipid-modifying therapy with adequate follow-up periods, typically in the order of 5 years, to fully define the clinical benefit of such therapy, particularly any cardiovascular mortality benefit [50,51,52,53,54,55].

## 6. Limitation of the Study

There are some limitations to the current study. The analysis performed is the first limitation and a prospective randomized trial is mandatory to confirm our preliminary results. Given the limited number of patients and the short follow-up period, we underline again that our study is a preliminary investigation, and the results must therefore be confirmed by an analysis on a larger sample.

The analysis refers to old papers because the treatment of dyslipidemia is a topic that has been discussed for a long time, and it is no coincidence that many of our references are more than 5 years old; nevertheless, all the latest articles and trials have been included. Although this is within the limitations of the study, our results must be considered significant because the drug has recently been placed on the market.

## 7. Conclusions

The use of Evolocumab in statin-resistant patients starting immediately after coronary artery bypass graft surgery significantly reduced LDL cholesterol and total cholesterol levels compared to statin treatment alone and showed its safety in the absence of major complications. However, at one year of follow-up, this result did not impact the reduction in major clinical events compared to other treatments.

## Figures and Tables

**Table 1:** Patients’ characteristics and laboratory values.

	Standard Treatment (n = 100)	Evo Treatment (n = 80)	*p*-Value
Age	66 ± 8	64 ± 8	0.1176
Gender	70 Males (70%)	60 Males (75%)	0.5641
Diabetes	80 no (80%)	61 no (76%)	0.6170
Smoking	88 no (88%)	63 no (78%)	0.1406
Insulin Therapy	93 no (93%)	75 no (93%)	1
Previous Myocardial infarction	90 no (90%)	71 no (88%)	0.9784
Previous PTCA	96 no (96%)	76 no (95%)	1
Previous CABG	98 no (98%)	79 no (98%)	1
Previous Stroke	98 no (98%)	78 no (97%)	1
Previous TIA	96 no (96%)	76 no (95%)	1
Hypertension	54 (no) 46 (yes)	51 no (63%)	0.2435
Total cholesterol mg/dL	Time 0: 191 ± 16 Time 1: 152 ± 13 Time 2: 130 ± 18	Time 0: 195 ± 13 Time 1: 111 ± 12 Time 2: 95 ± 8	0.0942 <0.001 <0.001
LDL cholesterolmg/dL	Time 0: 155 ± 16 Time 1: 114 ± 12 Time 2: 84 ± 20	Time 0: 157 ± 14 Time 1: 72 ± 12 Time 2: 44 ± 9	0.242 <0.001 <0.001
HDL cholesterol mg/dL	Time 0: 36 ± 5 Time 1: 37 ± 3 Time 2: 46 ± 8	Time 0: 37 ± 4 Time 1: 38 ± 2 Time 2: 51 ± 5	0.162 0.259 <0.001
Triglycerides mg/dL	Time 0: 179 ± 19 Time 1: 174 ± 17 Time 2: 172 ± 17	Time 0: 176 ± 17 Time 1: 165 ± 15 Time 2: 132 ± 18	0.289 <0.001 <0.001
Alanine Transaminase U/L	Time 0: 35 ± 5 Time 1: 39 ± 6 Time 2: 52 ± 18	Time 0: 37 ± 5 Time 1: 44 ± 7 Time 2: 62 ± 24	0.0054 <0.001 0.0040

## Data Availability

The original contributions presented in the study are included in the article; further inquiries can be directed to the corresponding author.
